# *Mycobacterium tuberculosis* complex Lineage 1: A neglected cause of tuberculosis

**DOI:** 10.1371/journal.pntd.0013513

**Published:** 2025-10-09

**Authors:** Venus Marie R. Rojas, Michaela Zwyer, Selim Bouaouina, Daniela Brites, Sonia Borrell, Sebastien Gagneux

**Affiliations:** 1 Swiss Tropical and Public Health Institute, Allschwil, Switzerland; 2 University of Basel, Basel, Switzerland; Tufts Medical Center, UNITED STATES OF AMERICA

## Abstract

The *Mycobacterium tuberculosis* complex (MTBC) phylogenetic lineages 1–4 (L1–L4) are the main causes of human tuberculosis (TB). Until now, most of the focus in the TB field has been on MTBC L2 and L4, as these two lineages are geographically widespread and have been repeatedly associated with multidrug resistance. By comparison, MTBC L1 has received little attention, partially because of its restricted geographical range that mainly includes low- to middle-income countries in South and Southeast Asia, and East Africa. However, recent estimates indicate that MTBC L1 is in fact the most common cause of human TB in terms of absolute numbers of TB patients, particularly among several high TB burden countries. As more L1 strains are being sampled in L1-endemic countries, the high genetic diversity of this geographically restricted MTBC lineage is slowly uncovered. This discovery has also impacted L1 nomenclature, which has been modified as new distinct L1 clades were identified. In parallel to the genomic discoveries ushered by progress in whole genome sequencing, clinical researchers have also studied several phenotypes that better describe L1 TB disease. L1 strains have been shown to have increased vulnerability to oxidative stress, which was associated with decreased virulence in animal and in vitro models. L1 infection also shows possible association with extrapulmonary TB and asymptomatic TB. However, despite belonging to the same lineage, L1 strains display phenotypic diversity that can be attributed to high within-lineage genetic diversity and possibly the interaction of different L1 genotypes with different human host genotypes. Among the clinical phenotypes that show heterogeneity are bacterial factors, immune profiles, and clinical virulence. The traditional view regarding the reduced transmissibility in L1 is now being challenged by new data indicating that L1 may be as transmissible as L2 or L4. Lastly, although historically referred to as being negatively associated with drug resistance, there is indication that the contribution of L1 to TB drug resistance is significant and that it may evolve drug resistance in ways distinct from those of other MTBC lineages.

## Introduction

With the end of the COVID-19 pandemic, tuberculosis (TB) is again the number one cause of human mortality due to a single infectious agent. An estimated 10.8 million new TB cases and 1.25 million deaths occurred in 2023 [1]. The outcome of TB infection and disease varies greatly, ranging from asymptomatic infection to pulmonary and extrapulmonary disease. Many patient and environmental factors are known to influence these variable outcomes. Increasingly, bacterial diversity is also being recognized as a contributing factor. TB in humans and other mammals is mainly caused by members of the *Mycobacterium tuberculosis* complex (MTBC). The MTBC comprises ten phylogenetic lineages adapted to humans and several clades adapted to different wild and domestic animals [2,3]. In human TB, most of the global burden is caused by L1, L2, L3, and L4, with L5–L10 together contributing only a minor proportion [4]. To date, most of the research on the biological and epidemiological consequences of MTBC diversity has been focusing on L2 and L4, largely because these two lineages are geographically widespread and have been associated with antibiotic resistance. By comparison, L1 and L3 have been neglected [5]. Several genomic epidemiology studies aiming to understand the evolution of drug resistance in TB disease use datasets of MTBC genomes that under-represent South and Southeast Asia, where most L1-endemic countries are [6,7]. Undersampling often occurs in L1-endemic countries due to logistical and financial difficulties of doing population-based sampling with limited resources. However, recent estimates indicate that L1 is the most common cause of human TB in terms of absolute numbers of patients affected. This is particularly true in several high TB burden countries of South- and Southeast Asia [4]. Here, we review and discuss the available literature on L1 and highlight some of the key characteristics that make L1 stand out from the other MTBC lineages.

## Methods

### The global population structure of MTBC L1

To determine the global population structure of MTBC L1, we used a global dataset of MTBC L1 strains to create a whole-genome-based phylogenetic tree and map the distribution of L1 sublineages around the world. This dataset comprised L1 genomes stored in our in-house database and included all genomes from countries where L1 is endemic [5,8,9]. In addition, this dataset also contained L1 genomes from TB low-burden countries such as those in North America and Europe. An in-house pipeline was used to extract nucleotide variants by mapping to a reference genome as previously reported [10]. The genomes included in the dataset were deemed of good quality based on the following criteria—coverage of at least 15× and showed no evidence of mixed lineage infection. Alignments of variable positions were used for creating a phylogenetic tree with RAxML v. 8.2.11 (options -m GTRCAT -V), using *Mycobacterium canettii* as an outgroup.

### Literature search

Pubmed and Ovid MEDLINE (ALL) searches were done for the purpose of this review. Different keywords were used, depending on the subtopic discussed. To broadly search about the global burden, population structure, and molecular epidemiology of MTBC L1, the following keywords were used—“tuberculosis” AND (“Lineage 1” OR “Indo-Oceanic” OR “EAI”) AND (“molecular epidemiology” OR “spoligotyping” OR “RFLP”). For the nomenclature of L1, the following keywords were used—“MTBC” AND (“sublineage” OR “barcode” OR “barcodes” OR “SNP”). For the different L1 phenotypes, the following keywords were used—“Tuberculosis” AND (“Lineage 1” OR “Indo-Oceanic” OR “EAI”) AND (“virulence” OR “immune profile” OR “transmission” OR “drug resistance” OR “extrapulmonary”). All database searches were supplemented by manual search done on the reference lists of each relevant publication.

## The global burden of MTBC L1

**Fig 1:**
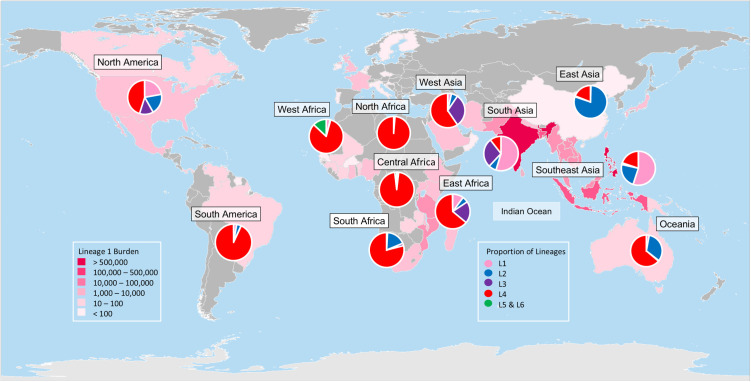
Estimated global burden of MTBC L1 and the proportion of different MTBC lineages worldwide. The pink heat map indicates the estimated absolute number of L1 TB cases in different countries, while the pie charts indicate the proportion of different MTBC lineages circulating in specific United Nations geoscheme geographical subregions. Data were previously published in a recent review [4]. The map used was created by Frank Bennett, Public Domain, via Wikimedia Commons (https://commons.wikimedia.org/wiki/File:BlankMap-World-Flattened.svg).

**Fig 2:**
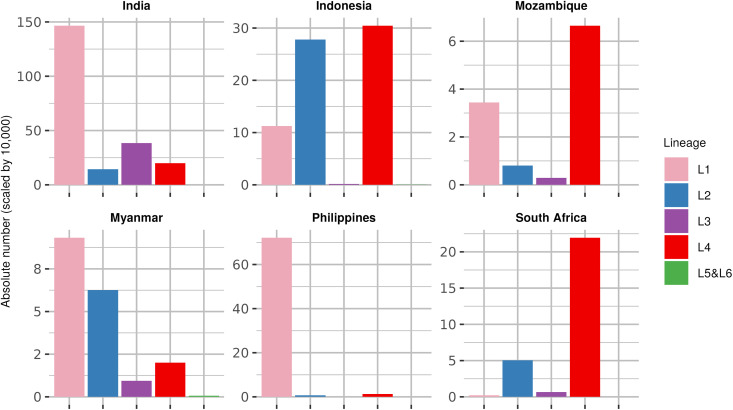
Estimated absolute number of cases of MTBC lineages circulating among L1-endemic high-burden TB, MDR/RR-TB, and TB-HIV countries. The absolute numbers on the y-axis are scaled by 10,000. The six countries indicated in the figure are L1-endemic countries that are also included in the WHO list of high-burden TB, MDR/RR-TB, and HIV/TB countries. The largest number of TB patients caused by L1 strains are in India and the Philippines, with approximately 1.4 million and 700,000 cases, respectively. Data used were obtained from the 2024 WHO Global TB Report [1] and a recently published review [4].

Compared to the globally widespread L2 and L4, L1 is geographically restricted. The geographical range of L1 is historically described as being around the rim of the Indian Ocean (Fig 1), namely in the regions of South Asia, Southeast Asia (SEA), and East Africa [5,8,9]. In a recently published review, the proportion estimates for the different MTBC lineages around the world was combined with the 2022 World Health Organization (WHO) global estimates for incident TB, which led to the estimate that L1 caused 2.8 million human TB cases in 2021, more than any other MTBC lineage. In contrast, L4 and L2 caused an estimated 2.5 and 1.3 million TB cases, respectively [4]. For this review, we defined L1-endemic countries as those found to have L1 genotypes consistently circulating and transmitting among individuals born in the respective countries. Using this definition, Brazil and some countries in West Africa and Oceania were considered to have L1-endemic strains, even though the total number of TB cases attributable to L1 are low relative to the other MTBC lineages circulating in these countries [5]. Some low-burden countries in Europe, North America and Australia, although not considered as L1-endemic countries, have high proportions of L1 cases, which, most likely reflect recent migrations from high-burden countries where L1 is endemic [11]. Importantly, many of the high-burden TB countries recognized by WHO are L1-endemic countries. These include India, the Philippines, Indonesia, Bangladesh, and Myanmar. Moreover, out of the ten countries at the intersection of the WHO list of high-TB, TB-HIV, and MDR/RR-TB burden [1], six are L1-endemic countries (Fig 2).

## The global population structure of L1

Using the SNP-based nomenclature originally published by Coll and colleagues [12], five L1 sublineages can be defined—L1.1.1, L1.1.2, L1.1.3, L1.2.1, and L1.2.2. In this review, we mainly discuss the L1 sublineages at this level of hierarchal subdivision, in line with the Coll nomenclature, which is the nomenclature most often used [12,13]. These sublineages may correspond to one or more of the following spoligotype groups—EAI1-SOM, EAI2-Manila, EAI2-Nonthaburi, EAI3-IND, EAI4-VNM, EAI5, EAI6-BGD1, EAI7-BGD2, EAI8-MDG, and Zero-copy [14]. Although widely used, the nomenclature by Coll and colleagues [12] has been revised and further expanded upon, which we will discuss in more detail below.

**Fig 3:**
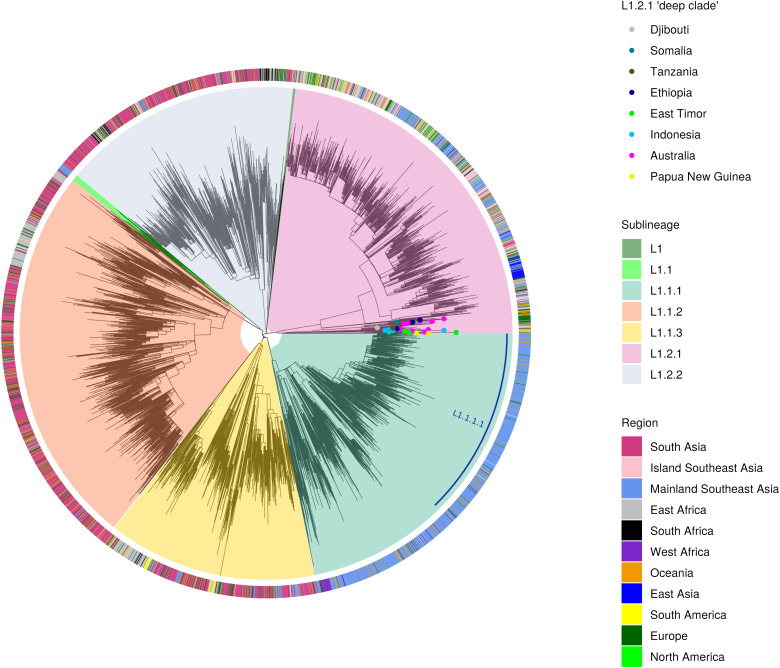
The global population structure of MTBC L1. The phylogenetic tree above consists of 4,171 MTBC L1 genomes originating from 63 countries. The phylogenetic tree indicates the sublineage classification according to L1 SNP barcodes published by Coll and colleagues [12]. Within L1.1.1, there is a clade designated as L1.1.1.1 (labeled in blue curved line). The genomes highlighted in light green (L1.1) and dark green (L1) are examples of genomes that could not be classified by Coll nomenclature, as previously published [5,15]. However, based on their position within the tree, they belong to L1.1.2 and L1.2.2, respectively. The strain’s geographical region of origin is based on the country of birth of the patient or if the country of birth is unknown, the country of isolation. The deeply branching genomes within the L1.2.1 clade, emphasized here with circular tip points, comprise strains from East Africa, Southeast Asia, and Oceania.

**Fig 4:**
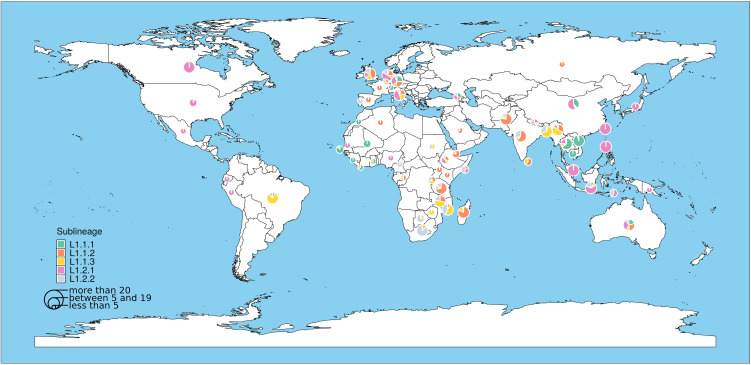
Global distribution of L1 sublineages. The MTBC genomes included here are the same as the ones represented in Fig 3. The genomes are classified according to the country of birth of the patient source of the isolate, or the country of isolation, when the country of birth is unknown. The size of the pie chart for each country is based on the number of genomes included in the dataset, with the smallest pie chart corresponding to less than five genomes and the largest with more than 20 genomes. The world map was generated from the R package “rnaturalearth” (https://CRAN.R-project.org/package=rnaturalearth).

The phylogenetic tree shown in Fig 3 is a representation of all major L1 sublineages from L1-endemic countries, based on previously reported data [8,11]. From this phylogenetic tree, one can appreciate that the global population of L1 is strongly phylogeographically structured, with some L1 sublineages being associated with particular geographical regions, as previously reported [5,15,16]. Specifically, most L1.1.1 sublineage strains occur in the mainland of Southeast Asia (SEA), which includes Vietnam, Thailand, Cambodia, and Laos. By contrast, in Island SEA, which includes the Philippines, Indonesia, Malaysia, Papua New Guinea, and East Timor, a high proportion of L1.2.1 can be observed. L1.1.2 on the other hand predominates in South Asia but is also seen in East Africa. L1.1.3 is prevalent in Bangladesh and Myanmar, as well as in East Africa, especially Malawi and Mozambique. Lastly, L1.2.2 is prevalent in South Asia, East Africa, and South Africa (Figs 3 and 4).

Outside of South Asia and SEA, the L1 strains identified in the West African countries of the Gambia, Ghana, Sierra Leone, and Mali belong to L1.1.1, which is the sublineage that predominates in Mainland SEA (Figs 3 and 4), suggesting that L1.1.1 was introduced to West Africa from SEA. However, the L1.1.1 sublineage is generally uncommon in the African continent, and the absolute number of TB cases due to L1.1.1 in West Africa is low compared to those caused by other MTBC lineages. In comparison, L1.1.2 is the most common L1 sublineage in East Africa; however, some strains from the East African countries of Ethiopia, Djibouti, Tanzania, and Somalia form a monophyletic clade within L1.2.1, which is genetically distinct from the L1.2.1 strains occurring in countries of Island SEA. This clade is deeply branching within L1.2.1 (Fig 3) and is a sister clade of another deeply branching clade that is composed of strains from Papua New Guinea and East Timor [5]. This deeply branching clade separated early in the evolutionary history of L1, and even though currently under-sampled, might provide information on the origin and the evolutionary forces that gave rise to the different clades of L1 we observe today. Finally, in the case of Brazil, genotyping methods based on spoligotyping and MIRU-VNTR have identified some L1 strains similar to the ones circulating in East Africa, South Africa, and South Asia—L1.1.2, L1.1.3, and L1.2.2, indicating that L1 in Brazil might have been introduced from Africa and/or South Asia [17,18] as further discussed below.

## The geographical origin of MTBC L1

To date, four studies have used different phylogeographical approaches to infer the most likely geographical origin of MTBC L1 [5,9,16,19]. Three of these studies used whole genome sequencing (WGS) data and inferred a South Asian origin for the L1 Most Recent Common Ancestor (MRCA, i.e., the population from which all existing L1 population descended from) [5,16,19]. One study used spoligotyping data and inferred a Southeast Asian origin for L1 [9]. Given the known limitations of spoligotyping compared to WGS [14,20], most of the current evidence thus supports a South Asian origin for L1. Both Mainland SEA and South Asia are regions where all five main L1 sublineages defined by Coll and colleagues [12] co-occur. However, the absolute number of L1 cases in South Asia is far greater than what is seen in Mainland SEA. Several researchers have studied the spread of L1 out of South Asia. One example is the introduction of L1.1.2 into Tanzania from South Asia about 300 years ago, which can likely be linked to Indian Ocean trade and migration [21]. Several minor dispersals of L1 from South Asia have also occurred to South America and West Africa, as reported from Brazil and several West African countries, possibly associated with the transatlantic slave trade [5,18]. Despite contention against SEA as the geographic origin of L1 MRCA, it likely played a role in the diversification and geographical spread of L1. This is particularly true for the MRCA of L1.1.1 and L1.2.1, which are thought to have originated in Mainland SEA and Island SEA and subsequently spread within their respective region and even to countries outside the region, such as in West and East Africa [5].

The importance of knowing the geographical origin of the MRCA within the context of MTBC infection is related to the observation first published more than 20 years ago that different MTBC strains are associated with certain human host populations [22]. This association persists even among individuals who migrate out of their birth country, regardless of being part of an epidemiologic cluster [23]. Furthermore, allopatric host–pathogen relationship, wherein the human host and the infecting MTBC pathogen originate from different geographic areas, shows lower infectivity specifically among geographically restricted lineages such as L1 [24]. Accordingly, we can say, for example, that an L1.2.1 strain is associated with individuals from island SEA (e.g., the Philippines) because the L1.2.1 MRCA originated in island SEA. Host–pathogen co-evolution or co-adaptation are among the mechanisms being explored to explain this observation [5,15,25].

## The evolving nomenclature of MTBC L1

The nomenclature of L1 has been changing through time, reflecting the changes in the genotyping methods used to classify MTBC strains. Older genotyping methods compared the presence or absence of genetic markers such as insertion sequences, direct repeats, and tandem repeats [26]. The current widely used nomenclature scheme, and the one mainly referred to in this review, is based on WGS. Thereby, single-nucleotide polymorphisms (SNPs) specific to different monophyletic clades are identified and can be used as SNP-barcodes to assign MTBC strains to the corresponding lineages and sublineages. Although WGS offers a high resolution for phylogenetic classification, SNP-barcoding has its limitations, particularly in the light of biased sampling. If the sampling is not able to capture all representative strains of a given lineage, some genetic diversity will remain unidentified. This is an issue for L1 in particular, since the capacity to conduct WGS remains limited in many L1-endemic countries. The recent inclusion of more WGS samples from L1-endemic countries allowed for finer delineation of various clades within L1 that were previously unidentified. For example, the observation that several L1 strains from Thailand could not be classified into any of the sublineages defined by the SNP-barcodes of Coll and colleagues [12] is a case in point [5,15] (see sublineages with light and dark green highlights in Fig 3). As a result, a re-evaluation of these SNP barcodes became necessary to accommodate smaller monophyletic groups or sublineages [27]. The major changes that occurred since the introduction of the Coll nomenclature include the subdivision of L1.2.1 into two sublineages—L1.2.1 and L1.2.2, and the reclassification of L1.2.2 into sublineages under the L1.3 clade [9,27,28]. Table 1 shows a summary of these recent changes in L1 sublineage nomenclature. The rationale used for supporting these changes in nomenclature included similarities in geographical location among strains in the newly defined clades, consistency with spoligotyping genotypes, smaller mean SNP distances within clades as opposed to between clades, inclusion of a minimum number of strains per clade, and having at least one SNP common within the clade that was not present outside of the clade [9,27,28]. However, caution is required when reading the clade numberings of the different nomenclature systems. Although these systems may use the same sublineage and clade numbers, they may not refer to the same L1 genotypes [9,27]. Moreover, with more comprehensive sampling and further WGS analysis of L1 strains in the future, more L1 diversity will likely be uncovered, and the corresponding nomenclature will continue to evolve.

**Table 1:** Comparison of different WGS-based SNP-typing schemes used to classify MTBC L1 strains.

	Coll (2014) [12]	Palittapongarnpim (2018) [15]	Napier (2020) [28]	Netikul (2022) [9]	Shitikov (2023) [27]
**No. of L1 isolates**	121	478	3,365	2,128	125^a^
**Countries included** **(** *N* **)**	Global (22)	Thailand (1)	Global (24)	Global (41)	Global (23)
**Changes in the nomenclature**	Further subdivision of L1 into five sublineages^b^	Expanded L1.1.2 to include strains that could not be classified by the previous L1.1.2 SNP barcodes Further delineation of L1.1.1 and L1.2.1 into smaller clades	Expansion of L1.1.1.1 clade Delineation of L1.1.3 into smaller clades Subdivision of Coll [12] sublineage L1.2.1 into L1.2.1 and L1.2.2 Delineation of a clade under L1.2.2 Defined L1.3.1 and L1.3.2, which was formerly Coll [12] sublineage L1.2.2	Further delineation of L1.1.1, L1.1.2, L1.1.3, and L1.2.2 into smaller clades	Proposed SNP-barcodes that unified both Napier [28] and Netikul [9] findings
**Additional clades named**	L1.1.1				
	L1.1.1.1	L1.1.1.2 to L1.1.1.9		L1.1.1.10 to L1.1.1.11	
	L1.1.2			L1.1.2.1 to L1.1.2.2	
	L1.1.3		L1.1.3.1 to L1.1.3.3	L1.1.3.4	
	L1.2.1^b^	L1.2.1.1 to L1.2.1.3			
	L1.2.2		L1.2.2.1 L1.3.1 to L1.3.2	L1.2.2.1 to L1.2.2.5	

^a^Out of 670 MTBC strains that represent five strains per genotype, which were chosen from the genotypes identified in an exploratory dataset of >10,000 MTBC strains.

^b^The previous SNP-typing systems [29,30] only identified the EAI-Manila clade, which correspond to the Coll [12] sublineage L1.2.1.

## Characterizing the genome diversity of MTBC L1

The genomic characteristics of L1 were often described in parallel to the developments in MTBC genotyping technologies. Historically, MTBC genotypes were defined based on the insertion sequence 6110 (IS6110) Restriction Fragment Length Polymorphism (RFLP) method. The range of IS6110 copy numbers across L1 strains is highly variable. Some L1 strains carry no copies at all, some have low numbers (1 or 2), and others can have as many as 15 copies [31]. Several studies have documented this phenomenon within individual countries or geographical regions [32–35]. The development of spoligotyping at the end of the 1990s complemented IS6110 RFLP-based typing. The East-African-Indian (EAI) spoligotyping family, which largely corresponds to L1, was first defined in 2001 [36], and is characterized by the absence of CRISPR spacers 29—32 and 34 (DVR 39—42, and DVR 44 in the spoligotype-43 format) and the presence of spacer 33 or DVR 43 in the spoligotype-43 format [37]. Shortly thereafter, the first so-called “large sequence polymorphisms” (LSPs), also known as “regions of difference” (RDs), were identified. Among these RDs, it was the discovery of TbD1 that allowed for the characterization of L1 as an “ancestral” or “ancient” lineage of the MTBC. L1 was described as “ancestral” to L2, L3, or L4, due to the presence of the TbD1 region, which was lost in the common ancestor of the three aforementioned “modern” lineages [38]. Given the absence of ongoing horizontal gene exchange in the MTBC [39], the presence of TbD1 most likely represents the ancestral state, which is also supported by the fact that TbD1 is present in all MTBC lineages except for L2, L3, and L4 [2,40,41]. The deletion of TbD1 therefore represents a phylogenetically derived state, which indicates a unique evolutionary event that occurred in the common ancestor of L2, L3, and L4 [2,40,41]. Eventually, by comparative genomics of larger MTBC strain collections, RD239 was found to be deleted in all L1 strains, which led to this lineage being referred to as the Indo-Oceanic lineage by the LSP-based nomenclature [22,42].

As WGS developed and became more widely used, many SNPs were discovered that were phylogenetically informative and specific for the different MTBC lineages and sublineages, allowing for the general SNP-based nomenclature of the MTBC used today. An additional advantage of WGS-typing is that it can be used to quantify the genetic distance between strains. Based on such measurements, we know that L1 has the highest within-lineage diversity among all the MTBC lineages. In an initial set of pairwise comparisons, the average genetic distance between 44 L1 MTBC strains obtained from a global dataset was 730 SNPs, as compared that of L4 (*n* = 64) and L2 (*n* = 37) strains, with an average genetic distance of around 600 and 200 SNPs, respectively [43]. A more recent comparison showed that the within-lineage diversity of L1 can be as high as 998 SNPs [9]. However, SNPs are not the only way of quantifying genetic distance. In fact, structural variants such as indels (e.g. RDs and LSPs) and repetitive sequences (e.g., direct repeats used in spoligotyping and insertion sequences such as IS6110) are ignored when measuring SNP distances using short-read WGS technology [44]. The genetic distance between strains could even be higher than what short-read WGS SNPs show, when considering all genetic variations together. Initial evidence from a recent MTBC pangenome study based on long-read sequencing showed that the diversity of L1 is high, as seen by the considerable genetic diversity of its accessory genome [45]. The high genetic diversity in L1 is not only due to diversification (or divergent evolution, as evidenced by phylogenetically informative SNPs and RDs) but is also due to homoplasy or convergent evolution (as evidenced by non-phylogentically informative RDs, such as RD3 and RD11, and similar spoligotype patterns in the direct repeat region among different L1 sublineages) [9,45,46].

## Connecting the MTBC L1 genotypes to phenotypes

How much of the MTBC genetic variation contributes to TB disease phenotype is an important question in TB clinical research. Unfortunately, it is one that is entangled with other factors such as host comorbidities and host-pathogen interaction [47–49]. Therefore, we present in this section of the review the important clinical phenotypes considered in TB disease and how it generally presents among L1 TB cases. However, the results of different studies are not always applicable to all human host populations. This is especially true for studies done *in vitro* or in animal models that lack many of the nuances of human TB disease [50].

### Experimental phenotypes

In 1963, Mitchison and colleagues observed that MTBC strains isolated from South Indian TB patients showed lower virulence in guinea pigs and increased susceptibility to reactive oxygen species when compared to MTBC isolates from British patients [51]. Follow-up studies around that time supported the notion that South Indian TB strains were indeed on average less virulent in guinea pigs and more susceptible to reactive oxygen stress than strains isolated from other countries [52]. Moreover, the lower virulence of these strains was associated with increased susceptibility to oxidative stress [53]. Even though increased susceptibility to oxidative stress in MTBC is not necessarily directly due to decreased virulence, it has been seen in other pathogenic bacteria that proteins involved in stress response also regulate virulence [54,55]. However, as mentioned previously, bacterial pathogenicity is not the only factor that dictates clinical virulence.

Today, we know that the large majority of the MTBC strains from South India belong to L1 and that endemic British MTBC isolates are mostly L4. A more recent study replicated the original experiments by Mitchison and colleagues using some of the same L1 isolates [56]. These authors established a possible role of TbD1 in MTBC virulence and used bacterial load as measurement of virulence. When comparing a wild-type L1 strain with an intact TbD1 region to a TbD1-deficient L1 strain, a higher bacterial load was observed in guinea pigs infected with the L1 mutant strain lacking TbD1. The authors also showed that the TbD1-deficient L1 strain was less susceptible to reactive oxygen and nitrogen species when compared to the wild-type strain [56]. Other experiments used spoligotyping information and compared EAI (i.e., L1) strains with non-EAI strains, in particular strains belonging to L2 and L4. Using MTBC strains isolated from Vietnam, one study measured the bacterial load in infected BALB/c mice and observed varying degrees of decreased virulence in L1 strains compared to either L2 or L4 [57]. In another study comparing strains isolated from Tanzania, L1 strains were shown to have a lower replication rate when compared to L2 in human monocyte-derived macrophages [58]. In summary, most (albeit limited) experimental evidence using infection models suggests L1 as being less virulent when compared to L2 and L4.

The inflammatory profile of L1 strains has also been studied to understand how the human immune system interacts with the different genetic variants of MTBC. In several studies, L1 induced higher TNF-α, IL-1β, and IL-12 levels in murine and human macrophages 24 and 48 hours after infection when compared to L2 and L4 [57,59,60]. Another study observed a wider range of values in the normalized median cytokine levels at 24 hours post-infection induced by L1 (*n* = 8), L5 (*n* = 1), and L6 (*n* = 7) strains, when compared to L2, L3, and L4 strains [60]. Other inflammatory cytokines that appear to be induced to a higher extent in L1 relative to L2, L3, and L4, 24–48 hours post-infection, include IL-6, IL-15, MIP-1a, CCL5, IL-8, and MCP-1 [59–61]. One study comparing L1 and L4 strains from Tanzania reported lower instead of higher inflammatory cytokines in L1 at 1, 4, and 7 days post-infection [58]. These somehow contradictory results may point to within-lineage phenotypic diversity among L1 strains, which would be consistent with the large genetic within-lineage diversity in L1 reviewed above. However, contradictory results on the inflammatory profile can also be simply due to the inherent limitations and differences in the experimental designs, such as varying multiplicities of infection (MOIs) upon infection among the different studies.

A few studies looked at the specific composition of the cell wall of L1 compared to other MTBC lineages. One study observed that L1 strains had a relatively higher abundance of alpha- and keto-mycolic acids when compared to L6, and relatively lower abundance of methoxy-mycolic acids compared to L2, L4, and L6 [62]. When examining a globally-representative collection of L1 strains, it was shown that the cell wall phenolic glycolipids varied across the L1 sublineages [13]. These authors also reported the presence of mycoside B in strains belonging to a subset of L1.2.2. This study is so far the first report of the presence of mycoside B in L1. Previously, mycoside B was reported in some strains belonging to L6 [13]. The fact that the L1 sublineages differ in their cell wall characteristics is further substantiated by a study demonstrating that strains belonging to L1.1.2 from India did not produce sulfolipids, while L1 strains from Vietnam did [63–65]. These findings on distinct MTBC cell wall phenotypes between L1 sublineages are important because many MTBC cell wall lipids are implicated in the host–pathogen cross-talk during TB infection and disease [66]. Finally, a recent study [67] used an *in vitro* granuloma-like infection model and found that L1 infections led to smaller granuloma formations when compared to L3 or L4.

### Clinical virulence

Despite the increasing evidence for differences in experimental phenotypes when comparing L1 to other MTBC lineages, only a few studies have explored L1 phenotypes in the clinic. Most studies to date have focused on other lineages (in particular L2) and included L1 into the larger group of “other lineages”, making any L1-specific conclusion difficult. However, one recent analysis on clinical outcomes in Vietnam based on 158 patient isolates found that L1.1.1.1 was associated with treatment failure and cavitary disease when compared to other lineages [68]. Similarly, a study analyzing 1,305 isolates from Thailand found that infection with strains from the Indo-Oceanic lineage (i.e., L1) was associated with increased mortality when compared to the East Asian lineage (i.e., L2) [69]. Clinical virulence in human pathogens like the MTBC, however, is not only a result of bacterial pathogenicity, but can also be affected by human host comorbidities and the interaction between the host immune system and the infecting MTBC genotype [49]. Hence, measures of experimental virulence *in vitro* and using other animal models are not always applicable to what we see clinically [50]. In summary, these limited clinical data so far are largely inconclusive and do not support a reduced clinical virulence due to L1, as could be expected based on the experimental data reviewed above.

### Extrapulmonary tuberculosis

While TB is primarily a pulmonary disease, TB can also affect many other parts of the human body. Collectively, extrapulmonary TB can represent more than 30% of cases in certain geographical settings like East Africa [70,71]. In addition to patient factors like co-infection with HIV and geographical region of origin [72–74], several findings suggest that L1 might also be associated with extrapulmonary TB. In particular, three studies analyzed large multinational collections of MTBC strains together with the associated clinical data and found L1 to be associated with extrapulmonary TB when compared to L2 [75,76], L3 [76], or L4 [76,77]. In contrast, another study from Germany showed no significant association between L1 and extrapulmonary TB [78]. In trying to understand the possible association between L1 and extrapulmonary TB, Saelens and colleagues [79] studied in much detail an L1 strain that caused an outbreak of extrapulmonary TB in the USA. The authors found that this L1 outbreak strain carried a full-length variant of the *esxM* gene that was truncated in strains belonging to L2, L3, and L4. They hypothesized that carrying full-length *esxM* could lead to a higher propensity of developing extrapulmonary TB due to morphological changes in the TB-infected macrophages that increase their motility. Of note, and similar to the TbD1 genomic region discussed above, the full-length version of *esxM* likely reflects the ancestral state while the truncated form reflects the derived state characteristic of all “modern” MTBC strains. Taken together, one could hypothesize that the “modern” strains might therefore have evolved to become more streamlined to cause pulmonary TB rather than extrapulmonary TB, when compared to L1 and to the other lineages exhibiting a full-length *esxM,* which potentially could enhance transmission in areas with lower TB incidence or lower population density.

### Transmission and infectiousness

Several genomic epidemiological studies have reported L1 strains to transmit less than other lineages (particularly L2 and L4) in both L1-endemic and non-endemic countries. These studies used genomic clustering rates based on specific SNP-distance thresholds and terminal branch lengths (TBLs) as proxies for recent transmission. A study from Vietnam reported reduced transmission of L1 strains compared to L2 and L4 [80]. One study in India and another in Tanzania also concluded L1 having a reduced transmission potential as compared to L2 and L4 [81], and L3 [21]. Several other studies that covered longer time periods or included larger sample sizes found that L1 strains were less likely to belong to transmission clusters [82–84]. SNP-based clustering is useful for detecting strains that are epidemiologically linked. However, there are some important limitations when using genetic clustering to infer differences in transmission. Among the confounding factors that affect using SNP-distances to infer transmissibility include differences in the molecular clock rate, the latency period between lineages, and the extent of sampling done (i.e., sampling period and sampling proportion) [85]. For example, a smaller cluster or a longer TBL can be due to any of the following—a longer latency period, a slower molecular clock rate, a shorter sampling period, or sampling of only a small proportion of cases [85]. By contrast, phylodynamic modeling can circumvent some of these limitations by simultaneously integrating evolutionary, demographic, and epidemiological parameters [10]. Phylodynamics is a statistical framework that integrates genetic data to estimate transmission parameters like the basic reproduction number (*R*_0_) and the effective reproduction number (*R*_e_). The values of *R*_0_ or *R*_e_ are more informative than TBLs or SNP clusters because they are validated measures of transmissibility that can also give an idea on the stability of an epidemic or the effectiveness of infection control measures [86]. While the value of *R*_0_ and *R*_e_ is determined by the sampling rate and transmission rate, TBLs are affected by many other factors mentioned above [85].

A study in Tanzania used such a phylodynamic approach and found that despite the reduced transmission rate for L1 compared to L2 and L3, there were no differences in the effective reproductive number (*R*_e_) between these lineages [21]. This phylodynamic model was recently further refined and used to analyze four independent MTBC genomic datasets from Malawi, Tanzania, The Gambia, and Vietnam [10]. The authors found that the time between transmission events was consistently longer in L1 and L6 compared to the “modern” L2, L3, and L4 in all four countries. These findings were attributed to a longer initial period of non-infectiousness (i.e., a delayed onset of infectious TB disease) in L1 and L6 compared to the modern lineages. Interestingly, in contrast to the results of SNP-based clustering and TBLs, the transmission rates in L1, L2, L3, and L4 inferred by this phylodynamic model were similar [10]. Taken together, while several genomic epidemiological studies suggest that L1 shows a reduced transmission potential, more sophisticated phylodynamic analyses show a more complex picture. Hence, the notion of L1 having reduced transmissibility might be too simplistic, which is also supported by the observation that in most L1-endemic countries, there is no evidence for L1 being outcompeted by other lineages over time [87]. Instead, L1, and probably some of the other MTBC lineages still retaining intact TbD1 and *esxM* versions, might differ in their evolutionary strategy in more subtle ways. Specifically, one could hypothesize that a slower progression from infection to disease with overt clinical symptoms is associated with a delayed infectious period. Some support for this hypothesis comes from a recent study in Canada were an association between L1 and asymptomatic TB has been reported in two independent cohorts [88]. The high population density in many L1-endemic countries may also allow L1 to transmit despite the presumed delay in onset of infectiousness. It has been noted that TB re-infection can occur especially in areas with high TB incidence [89,90]. This also supports the notion that in densely populated L1-endemic countries, there is a constant supply of susceptible hosts, which allows for continuous infection and transmission. In a modeling done on TB disease using parameters that are more attuned with endemic TB communities, re-exposure was seen to increase the probability of entering an infectious state [91].

## Drug resistance in MTBC L1

Multidrug-resistant TB (MDR-TB) has repeatedly been associated with L2 [92]. By contrast, the contribution of L1 in the MDR-TB epidemic remains unclear. This is important in light of the fact that among the ten countries with the highest absolute incidence number of MDR-TB, seven are L1-endemic countries—India, Indonesia, the Philippines, Pakistan, Myanmar, South Africa, and Vietnam [1]. However, the characteristics of MDR L1 strains have rarely been investigated. Several studies have shown that isoniazid resistance encoded by mutations in the *inhA* promotor located in the *fabG1* (*mabA*) gene are overrepresented in L1 strains compared to L2 or L4 [93–95]. The reason underlying the association between L1 and *fabG1* drug resistance mutations is unknown, but could be related to the overall higher susceptibility of L1 to oxidative stress [51,53], making the catalase-peroxidase activity encoded by *katG* particularly essential for intracellular detoxification in L1-infected host cells. Therefore, it may be more beneficial for L1 to minimize *katG* mutations, which gives preference towards other mutations that confer isoniazid resistance. As for rifampicin, the mutation *rpoB* S450L is globally the most frequent cause of resistance. Based on the WHO MTBC Catalog of Drug Resistance Mutations, out of around 16,000 isolates with rifampicin resistance, 64% had an *rpoB* S450L mutation [96]. In the Philippines, however, where most of the circulating MTBC strains are L1, only around 40% of rifampicin-resistant MTBC strains harbored this mutation [97]. A similar observation was reported in India [98]. These findings suggest that in L1, other mutations in the *rpoB* gene or in genes other than **rpoB*,* might play an important role in rifampicin resistance. During the process of developing a standardized protocol for routine phenotypic drug susceptibility testing for the new anti-TB drug pretomanid, it was noticed that L1 strains exhibited a higher minimal inhibitory concentration (MIC) for pretomanid when compared to L2, L3, or L4 strains [99]. This increased MIC for pretomanid in L1 was confirmed in a later study [100]. However, the clinical implications of this for the treatment of MDR-TB in L1-endemic countries remain to be determined [100]. Conversely, it has been reported that L1 had a reduced MIC to bedaquiline [68], which may have a beneficial effect on the treatment of patients infected with L1. This may be related to the observation of a loss-of-function (LOF) mutation in the *mmpL5* gene of strains from sublineage L1.1.1.1 [101]. This *mmpL5* LOF mutation is thought to increase sensitivity to the new drug bedaquilin [68]. Another LOF mutation was identified in the *whiB7* gene among strains belonging to sublineage L1.2.1 [102]. The *whiB7* gene has previously been shown as partially responsible for the intrinsic resistance of MTBC to many drugs such as the macrolides class of antibiotics [103]. The LOF mutation discovered in this gene resulted in sensitivity to clarithromycin, opening the door for alternative, bacterial genotype-specific treatments for TB [102].

## Conclusions

Despite being the most common cause of human TB in terms of absolute numbers, we still have an incomplete understanding of the biology and epidemiology of MTBC L1. While experimental infection studies suggest a reduced virulence of L1 strains compared to strains belonging L2, L3, and L4, the limited clinical data available draws a more complex picture. Moreover, the repeated association of L1 with extrapulmonary TB, the delayed progression to the infectious disease state indicated by phylodynamic modeling, and the association with asymptomatic TB, are suggestive of distinct life history traits of L1 compared to the other human-adapted MTBC lineages. More research is necessary to understand the intersection between the human host and L1. These studies should especially be placed into the context of the environment in L1-endemic countries, where socioeconomic factors play important roles in shaping the health of individuals at risk for or already infected with MTBC.

Finally, the fact that L1 is the most genetically diverse MTBC lineage with a strong phylogeographical population structure might reflect local adaptation of L1 genotypes to different human host populations. However, more work is needed to explore this hypothesis. Beyond determining the evolutionary forces that resulted in an increased genetic diversity within L1, we also need to mechanistically study the functional consequences of this diversity. In light of the ongoing efforts to develop improved TB vaccines [104] and new anti-TB treatments [105], care should be taken to ensure a broad efficacy, including against infection and disease caused by L1.

**Box 1. Key learning points**

a. MTBC L1 is globally the most frequent cause of human TB in terms of the estimated absolute number of patients affected, specifically among the high-burden TB countries India, the Philippines, Indonesia, and Bangladesh.
b. The global population of MTBC L1 is highly phylogeographically structured, with the different L1 sublineages showing particular geographical associations.
c. Increase in sampling of MTBC strains from L1-endemic countries resulted in the identification of new L1 genotypes. More L1 diversity will likely be uncovered in the future.
d. In addition to being the most genetically diverse MTBC lineage, L1 strains also exhibit large phenotypic diversity, the relevance of which for global TB control remains to be established.
e. Differences in transmission dynamics and progression to disease in L1 compared to L2, L3, and L4 suggest subtle differences in life history traits across MTBC lineages.

**Box 2. Five key references**

a. Goig GA, Windels EM, Loiseau C, Stritt C, Biru L, Borrell S, et al. Ecology, global diversity and evolutionary mechanisms in the *Mycobacterium tuberculosis* complex. Nature Reviews Microbiology. 2025;23:602–14. doi: https://doi.org/10.1038/s41579-025-01159-w.
b. Bottai D, Frigui W, Sayes F, Di Luca M, Spadoni D, Pawlik A, et al. TbD1 deletion as a driver of the evolutionary success of modern epidemic *Mycobacterium tuberculosis* lineages. Nature Communications. 2020;11(1):684. https://doi.org/10.1038/s41467-020-14508-5.
c. Saelens JW, Sweeney MI, Viswanathan G, Xet-Mull AM, Jurcic Smith KL, Sisk DM, et al. An ancestral mycobacterial effector promotes dissemination of infection. Cell. 2022;185(24):4507–25.e18. doi: https://doi.org/10.1016/j.cell.2022.10.019.
d. Netikul T, Thawornwattana Y, Mahasirimongkol S, Yanai H, Maung HMW, Chongsuvivatwong V, et al. Whole-genome single nucleotide variant phylogenetic analysis of *Mycobacterium tuberculosis* Lineage 1 in endemic regions of Asia and Africa. Scientific Reports. 2022;12(1):1565. https://doi.org/10.1038/s41598-022-05524-0.
e. Windels EM, Valenzuela Agüí C, de Jong BC, Meehan CJ, Loiseau C, Goig GA, et al. Onset of infectiousness explains differences in transmissibility across *Mycobacterium tuberculosis* lineages. Epidemics. 2025;51:100821. doi: https://doi.org/10.1016/j.epidem.2025.100821.
